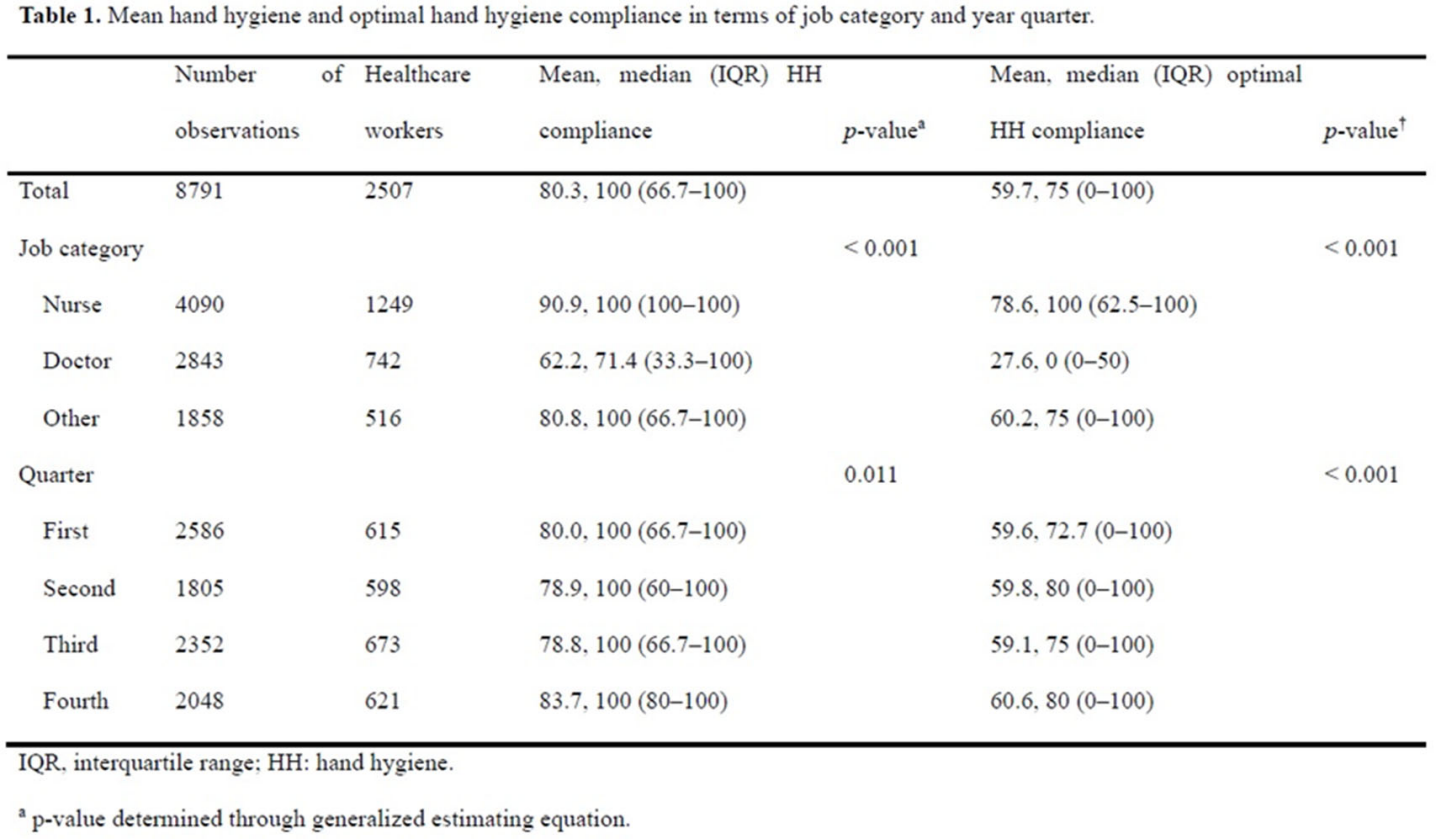# Appropriate Number of Observations to Determine Hand Hygiene Compliance Among Healthcare Workers

**DOI:** 10.1017/ash.2021.125

**Published:** 2021-07-29

**Authors:** Se Yoon Park, Eunjung Lee, Suyeon Park, Tae Hyong Kim, Sungho Won

## Abstract

**Background:** We sought to determine the minimum number of observations needed to determine hand hygiene (HH) compliance among healthcare workers. **Methods:** The study was conducted at a referral hospital. We retrospectively analyzed the result of HH monitoring from January to December 2018. HH compliance was calculated by dividing the number of observed HH actions by the total number of opportunities. Appropriate HH compliance rates were calculated based on the 6-step technique, modified from the World Health Organization (WHO) recommendation. The minimum number of required observations (n) was calculated by the following equation using overall mean value (r), absolute precision (d), and confidence interval (1-α) [The equation: n^3^ Zα/22×ρ×1-ρ/d2]. We considered ds of 5%, 10%, 20%, and 30%, with CIs of 99%, 95%, and 90%, respectively. Among the various cases, we focused on 10% for d and 95% for CI. **Results:** During the study period, 8,791 opportunities among 1,168 healthcare workers were monitored. The mean HH compliance and appropriate HH compliance rates were 80.3% and 59.7%, respectively (Table 1). The minimum number of observations required to determine HH compliance rates ranged from 2 (d, 30%; CI, 90%) to 624 (d, 5%; CI, 99%), and the minimum number of observations for optimal HH compliance ranged from 5 (d, 30%, CI, 90%) to 642 (d, 5%; CI, 99%) (Figure 1). At 10% absolute precision with 95% confidence, the minimum number of observations to determine HH and optimal HH compliance were 61 and 92, respectively. **Conclusions:** The minimum number of observations to determine HH compliance varies widely according to setting, but at least 5 were needed to determine optimal HH compliance.

**Funding:** No

**Disclosures:** None

**Figure 1.**
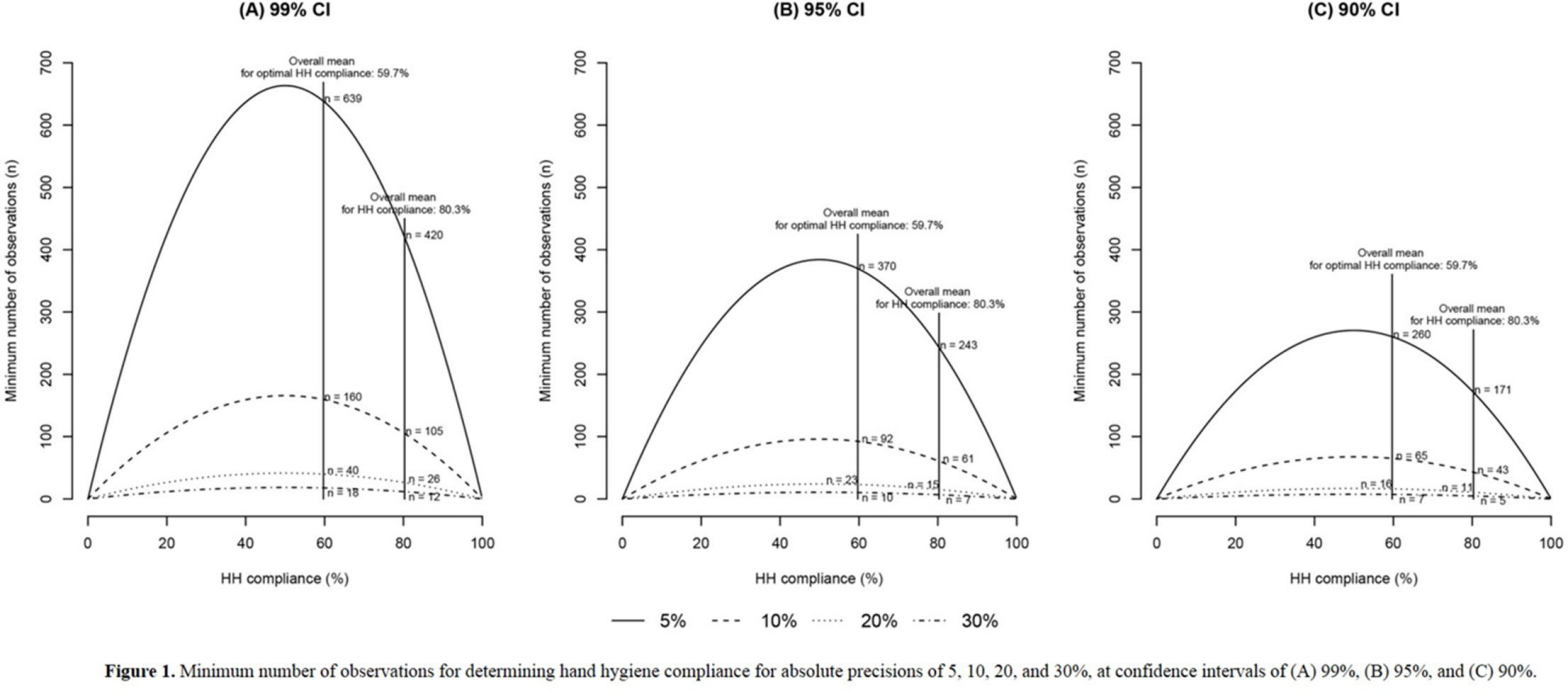

**Table 1.**